# Adverse cardiac remodeling augments adipose tissue ß-adrenergic signaling and lipolysis counteracting diet-induced obesity

**DOI:** 10.1016/j.jbc.2023.104788

**Published:** 2023-05-05

**Authors:** Stephanie Kolleritsch, Laura Pajed, Anna Tilp, Victoria Hois, Isabella Pototschnig, Benedikt Kien, Clemens Diwoky, Gerald Hoefler, Gabriele Schoiswohl, Guenter Haemmerle

**Affiliations:** 1 Institute of Molecular Biosciences, University of Graz, Graz, Austria; 2 Division of Endocrinology and Diabetology, Medical University of Graz, Graz, Austria; 3 Diagnostic & Research Institute of Pathology, Medical University of Graz, Graz, Austria; 4 BioTechMed, Graz, Graz, Austria; 5 Department of Pharmacology and Toxicology, University of Graz, Graz, Austria

**Keywords:** adipose tissue, cardiac hypertrophy, lipolysis, Perilipin 5, thermogenesis

## Abstract

Cardiac triacylglycerol accumulation is a common characteristic of obesity and type 2 diabetes and strongly correlates with heart morbidity and mortality. We have previously shown that cardiomyocyte-specific perilipin 5 overexpression (Plin5-Tg) provokes significant cardiac steatosis *via* lowering cardiac lipolysis and fatty acid (FA) oxidation. In strong contrast to cardiac steatosis and lethal heart dysfunction in adipose triglyceride lipase deficiency, Plin5-Tg mice do not develop heart dysfunction and show a normal life span on chow diet. This finding prompted us to study heart function and energy metabolism in Plin5-Tg mice fed high-fat diet (HFD). Plin5-Tg mice showed adverse cardiac remodeling on HFD with heart function only being compromised in one-year-old mice, likely due to reduced cardiac FA uptake, thereby delaying deleterious cardiac lipotoxicity. Notably, Plin5-Tg mice were less obese and protected from glucose intolerance on HFD. Changes in cardiac energy catabolism in Plin5-Tg mice increased ß-adrenergic signaling, lipolytic, and thermogenic protein expression in adipose tissue ultimately counteracting HFD-induced obesity. Acute cold exposure further augmented ß-adrenergic signaling in Plin5-Tg mice, whereas housing at thermoneutrality did not protect Plin5-Tg mice from HFD-induced obesity albeit blood glucose and insulin levels remained low in transgenic mice. Overall, our data suggest that the limited capacity for myocardial FA oxidation on HFD increases cardiac stress in Plin5-Tg mice, thereby stimulating adipose tissue ß-adrenergic signaling, triacylglycerol catabolism, and thermogenesis. However, long-term HFD-mediated metabolic stress causes contractile dysfunction in Plin5-Tg mice, which emphasizes the importance of a carefully controlled dietary regime in patients with cardiac steatosis and hypertrophy.

Obesity exerts a strong impact on heart energy metabolism and highly correlates with cardiac morbidity and mortality (1). An increase in cardiac fatty acid (FA) flux and uptake in obesity (or type 2 diabetes) eventually exceeds the cardiomyocyte’s capacity for FA oxidation and/or FA esterification to generate neutral lipids like triacylglycerols (TAGs) and cholesteryl esters. High levels of nonesterified FAs can directly disturb the cellular integrity or indirectly provoke organellar dysfunction *via* the generation of potentially harmful lipids like ceramides and diacylglycerols, altogether referred to as lipotoxicity (2). Several transgenic mouse models have been generated to study the impact of cardiac lipotoxicity and steatosis on cardiac energy metabolism and function. Mice with cardiomyocyte-specific overexpression of long-chain acyl coenzyme A synthetase (3) or peroxisome proliferator-activated receptor (PPAR)α (4) exhibit massive cardiac FA uptake and TAG accumulation eventually leading to cardiac hypertrophy and lethal left ventricular (LV) dysfunction. To counteract the development of lipotoxic heart dysfunction several strategies have been pursued including the reduction of cardiac FA shuttling *via* inhibition of adipose tissue lipolysis (5, 6, 7) or lowering cardiac FA oxidation, thereby increasing glucose utilization and consequently lowering cardiac FA flux (8). However, lowering cardiomyocyte FA oxidation or increasing FA neutralization within the cardiac TAG pool as strategy to counteract the progression of heart disease was challenged by the severe and early lethal LV dysfunction of mice lacking adipose triglyceride lipase (ATGL) (9). Mice lacking ATGL develop severe cardiac steatosis functionally linked to defective PPARα signaling and impaired mitochondrial FA oxidation, which can be reversed upon administration of a PPARα agonist (10). Similarly, humans carrying *ATGL* loss-of-function mutations develop severe and life-threatening cardiomyopathy eventually requiring heart transplantation at an early age (11). Efficient ATGL-mediated TAG hydrolysis depends on the interaction with its coactivator comparative gene identification-58 (CGI-58; also known as alpha/beta hydrolase domain containing 5). Mice lacking CGI-58 in muscle also exhibit massive cardiac TAG accumulation and develop lethal heart dysfunction albeit life span is significantly increased compared to ATGL-deficient mice (12). Furthermore, cardiac lipolysis is under the regulation of perilipin 5 (PLIN5) (13), which interacts with CGI-58 at the lipid droplet under nonstimulated conditions. Upon ß-adrenergic stimulation and PKA-mediated PLIN5 phosphorylation, this interaction is resolved thereby allowing CGI-58 to stimulate ATGL lipolytic activity (14, 15). We and others have shown that cardiomyocyte-specific PLIN5 overexpression (Plin5-Tg) provokes significant TAG accumulation in cardiac muscle *via* lowering lipolysis and FA oxidation (16, 17). Unexpectedly, and despite a similar degree of cardiac fat accumulation compared to ATGL deficiency, Plin5-Tg mice show a normal life span and are protected from lipotoxic cardiomyopathy and LV dysfunction likely *via* maintaining the metabolic flexibility of cardiomyocytes and increased expression of genes protecting from oxidative stress (18). These findings prompted us to study the impact of high-fat diet (HFD)-induced metabolic stress on heart function and energy metabolism in Plin5-Tg mice. We show that Plin5-Tg mice are still protected from the development of heart failure albeit the cardiac function declines with age likely due to prolonged exposure to metabolic stress. Of particular interest, Plin5-Tg mice were protected from diet-induced obesity in association with improved insulin sensitivity and glucose tolerance. Functionally, augmented adipose tissue sympathetic innervation followed by increased ß-adrenergic signaling and thermogenic activity counteracted the development of HFD-induced obesity in Plin5-Tg mice.

## Results

### Altered cardiac energy metabolism in Plin5-Tg mice fed HFD

**Figure 1:**
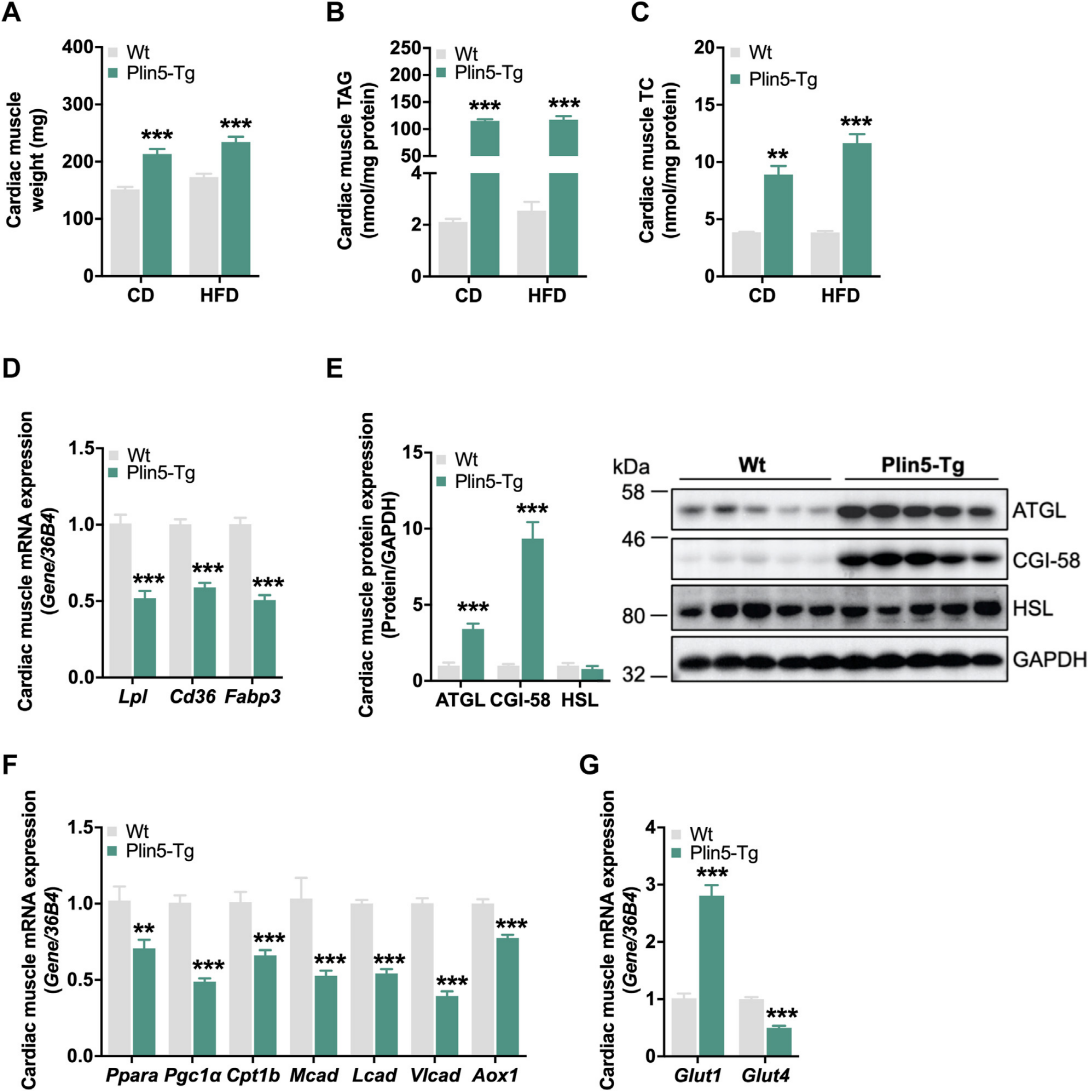
**Cardiac energy metabolism in Plin5-Tg micefed HFD.***A*, heart weights, (*B*) triacylglycerol (TAG), and (*C*) total cholesterol (TC) levels on chow diet (CD) and high-fat diet (HFD; 30 weeks, *ad libitum* fed, n = 4–5). *D*, cardiac mRNA expression of genes involved in fatty acid (FA) uptake relative to *36B4* reference gene determined by qPCR with WT mice arbitrarily set to 1 for each gene. *E*, cardiac protein expression of lipolytic proteins. *Left*: quantification of ATGL, CGI-58, and HSL relative to GAPDH (26 weeks, *ad libitum* fed, n = 5). *Right*: representative immunoblots. *F*, cardiac mRNA expression of genes involved in FA oxidation and (*G*) *Glut1* and *Glut4* mRNA expression relative to *36B4* reference gene determined by qPCR with WT mice arbitrarily set to 1 for each gene (26 weeks, *ad libitum* fed, n = 6). Data are presented as means ± SEM. Statistical significance was determined using unpaired Student's *t* test (∗∗*p* < 0.01; ∗∗∗*p* < 0.001 for effect of genotype). ATGL, adipose triglyceride lipase; CGI, comparative gene identification; FA, fatty acid; Glut, glucose transporter; HFD, high-fat diet; HSL, hormone-sensitive lipase; qPCR, quantitative PCR.

Since Plin5-Tg mice do not develop LV dysfunction on a standard chow diet despite massive cardiac TAG accumulation (16, 17), we examined cardiac energy metabolism and heart function in Plin5-Tg mice during HFD-induced metabolic stress. PLIN5 protein levels were equally increased in cardiac muscle of Plin5-Tg mice either fed chow or HFD (Fig. S1*A*). As expected, heart weight (Fig. 1*A*), cardiac TAG, and total cholesterol levels (Fig. 1, *B* and *C*) were significantly elevated in HFD-fed Plin5-Tg compared to WT mice, but comparable to Plin5-Tg mice–fed chow diet. mRNA expression of genes involved in FA uptake including *Lpl, Cd36*, and *Fabp3* was reduced in Plin5-Tg mice–fed HFD (Fig. 1*D*), indicating that cardiac steatosis was not further aggravated by increased FA uptake on HFD. As previously shown (16), ATGL and CGI-58 but not hormone-sensitive lipase (HSL) protein expression was significantly upregulated in cardiac muscle of Plin5-Tg mice (Fig. 1*E*), most likely an adaptation to impaired lipolysis upon PLIN5 overexpression. We have previously shown that cardiac lipolysis is required for PPARα and PGC1α target gene expression (10). In accordance with low cardiac lipolysis in Plin5-Tg mice (16), mRNA expression of *Pparα*, *Pgc1α*, and PPARα target genes including *carnitine palmitoyltransferase 1b*, *acyl-coA* *dehydrogenases* (*Mcad, Lcad, Vlcad*), and *acyl-coA oxidase* was significantly reduced in cardiac muscle of HFD-fed Plin5-Tg mice compared to controls (Fig. 1*F*). In contrast, mRNA expression of the insulin-independent *glucose transporter 1* (*Glut1*) was substantially upregulated, whereas *Glut4* expression was decreased (Fig. 1*G*). Insulin-mediated phosphorylation of AKT S473 was comparable in cardiac muscle of both genotypes (Fig. S1*B*). Thus, and as already demonstrated in transgenic mice on chow diet (16), Plin5-Tg mice–fed HFD likely switch toward increased insulin-independent glucose uptake and lower FA catabolism in cardiac muscle.

### Cardiac hypertrophy and moderate heart dysfunction in Plin5-Tg mice fed HFD

**Figure 2:**
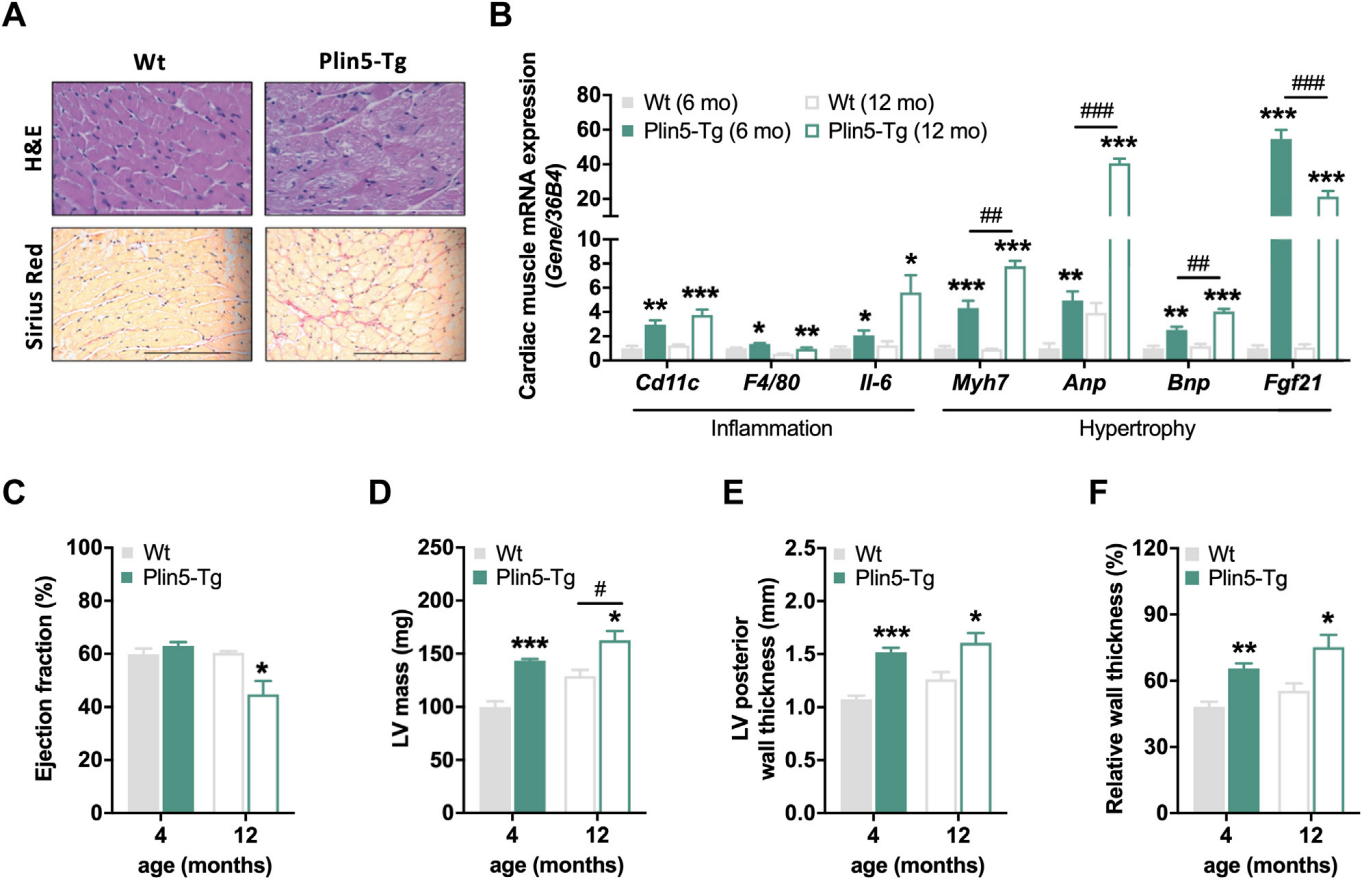
**Cardiac hypertrophy and moderate heart dysfunction in Plin5-Tg micefed HFD.***A*, representative histological images of heart sections stained with H&E and Sirius red. The scale bar represents 200 μm (26 weeks, *ad libitum* fed). *B*, cardiac mRNA expression of immune cell markers as well as genes involved in inflammation and hypertrophy of 6- and 12-month-old mice relative to *36B4* reference gene determined by qPCR with WT mice arbitrarily set to 1 for each gene (26 weeks & 52 weeks, *ad libitum* fed, n = 6). *C*–*F*, morphologic and functional cardiac parameters of 4- and 12-month-old mice by MRI: (*C*) ejection fraction, (*D*) *left ventricular* (LV) mass, (*E*) LV posterior wall thickness, and (*F*) relative wall thickness (*ad libitum fed*, n = 4–6). Data are presented as means ± SEM. Statistical significance was determined using unpaired Student's *t* test (∗*p* < 0.05; ∗∗*p* < 0.01; ∗∗∗*p* < 0.001 for effect of genotype. ^#^*p* < 0.05; ^##^*p* < 0.01; ^###^*p* < 0.001 for effect of age). HFD, high-fat diet; LV, left ventricular; qPCR, quantitative PCR.

Cardiac TAG accumulation is often linked to cardiac fibrosis and inflammation. Hearts from HFD-fed Plin5-Tg mice exhibited increased cardiac fibrosis shown by pronounced Sirius red staining of cardiac tissue sections (Fig. 2*A*). mRNA expression of immune cell markers as well as inflammatory and hypertrophic genes including *Cd11c*, *F4/80*, *interleukin-6*, *myosin heavy chain beta* , atrial natriuretic peptide (*Anp*), brain natriuretic peptide *(Bnp*), and *fibroblast growth factor 21* (*Fgf21*) was increased in Plin5-Tg mice compared to WT mice (Fig. 2*B*). The rise in hypertrophic gene expression (*myosin heavy chain beta, Anp*, and *Bnp*) was further pronounced in 1-year-old transgenic mice. MRI revealed a similar ejection fraction of Plin5-Tg mice compared to controls at the age of 4 months (Fig. 2*C*) despite a signature of cardiac hypertrophy and adverse cardiac remodeling including an increase in LV mass, LV posterior wall thickness, and relative wall thickness in Plin5-Tg mice (Fig. 2, *D–F*). However, in 1-year-old transgenic mice, ejection fraction declined (Fig. 2*C*) together with a further increase in LV mass (Fig. 2*D*). Thus, low cardiac lipolysis in HFD-fed Plin5-Tg mice does not acutely provoke heart dysfunction but deteriorates heart function with age in the presence of sustained HFD-induced metabolic stress.

### Increased adipose tissue ß-adrenergic signaling counteracts diet-induced obesity in Plin5-Tg mice

**Figure 3:**
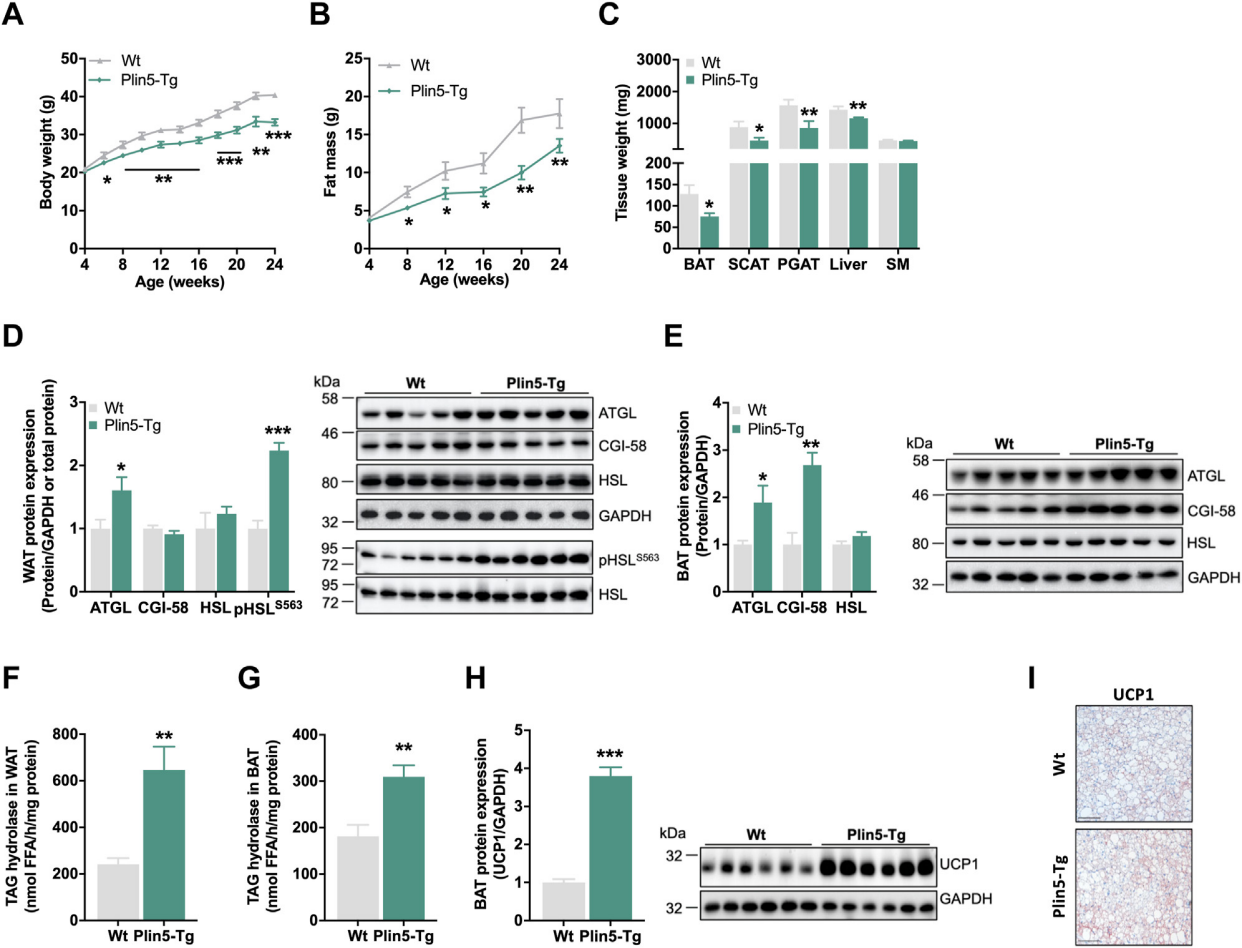
**Increased lipolytic activity counteracts diet-induced obesity in Plin5-Tg micefed HFD.***A*, longitudinal body weight and (*B*) total fat mass (4–26 weeks, *ad libitum* fed, n = 8–10). *C*, tissue weights (28 weeks, *ad libitum* fed, n = 4–5). *D*, protein expression of lipolytic proteins in WAT (perigonadal AT). *Left*: quantification of ATGL, CGI-58, and HSL relative to GAPDH and phospho-HSL^S563^ relative to total HSL (26 weeks, *ad libitum* fed, n = 5–6). *Right*: representative immunoblots. *E*, protein expression of lipolytic proteins in BAT. *Left*: quantification of ATGL, CGI-58, and HSL relative to GAPDH (30 weeks, *ad libitum* fed, n = 5). *Right*: representative immunoblots. Triacylglycerol (TAG) hydrolase activity in (*F*), WAT (perigonadal AT) and (*G*), BAT determined by [^3^H]-labeled oleic acid release (26 weeks, *ad libitum* fed, n = 6). *H*, protein expression of UCP1 in BAT. *Left*: quantification relative to GAPDH (30 weeks, *ad libitum* fed, n = 6). *I*, representative images of UCP1 immunostaining in BAT. The scale bar represents 100 μm (30 weeks, *ad libitum* fed). Data are presented as means ± SEM. Statistical significance was determined using unpaired Student's *t* test (∗*p* < 0.05; ∗∗*p* < 0.01; ∗∗∗*p* < 0.001 for effect of genotype). AT, adipose tissue; ATGL, adipose triglyceride lipase; BAT, brown adipose tissue; CGI, comparative gene identification; HFD, high-fat diet; HSL, hormone-sensitive lipase; PGAT, perigonadal AT; SCAT, subcutaneous AT; SM, skeletal muscle (quadriceps); UCP1, uncoupling protein 1; WAT, white adipose tissue.

Unexpectedly, and in contrast to Plin5-Tg mice on chow diet (16, 17), transgenic mice–fed HFD gained less body weight and fat mass than WT mice starting at the age of 8 weeks (Fig. 3, *A* and *B*). Despite these changes, food intake, locomotor activity, and oxygen consumption rate were comparable between genotypes (Fig. S2, *A–C*). Plasma lipids including FAs, TAGs, and ketone bodies were similar between WT and Plin5-Tg mice irrespective of the feeding status, whereas glycerol levels were significantly increased (1.3-fold) in fasted transgenic mice (Table 1). Reduced body weight and fat mass were accompanied by lower tissue weight of brown adipose tissue (BAT) and white adipose tissue (WAT: subcutaneous and perigonadal adipose tissue as well as the liver (Fig. 3*C*). Since reduced adipose tissue mass may be a consequence of increased lipolytic capacity, we determined the expression and activation of lipolytic enzymes in WAT (perigonadal adipose tissue) and BAT. In agreement, ATGL protein expression and phosphorylation of HSL S563 in WAT (Fig. 3*D*) as well as ATGL and CGI-58 protein expression in BAT were upregulated in Plin5-Tg mice (Fig. 3*E*). In line with increased expression and/or phosphorylation of lipolytic proteins, Plin5-Tg mice showed increased TAG hydrolase activity in adipose tissue (Fig. 3, *F* and *G*). In BAT, the increased lipolytic response of Plin5-Tg mice was accompanied by higher uncoupling protein 1 (UCP1) protein levels (Fig. 3, *H* and *I*), suggesting increased uncoupling and heat production. The presence of smaller lipid droplets within brown adipocytes of Plin5-Tg mice (Fig. S2*D*) was in accordance with increased BAT lipolysis.

**Table 1:** Blood and plasma parameters of Plin5-Tg and WT mice on HFD

Parameters		WT	Plin5-Tg
FA [mM]	*ad libitum* fed	0.25 ± 0.03	0.29 ± 0.03
	fasted	0.76 ± 0.04	0.79 ± 0.04
TAG [mM]	*ad libitum* fed	0.62 ± 0.03	0.77 ± 0.06
	fasted	0.96 ± 0.07	1.06 ± 0.08
Glycerol [mM]	*ad libitum* fed	0.28 ± 0.03	0.34 ± 0.03
	fasted	0.33 ± 0.03	0.44 ± 0.02∗
Ketone bodies [mM]	*ad libitum* fed	0.18 ± 0.00	0.20 ± 0.01
	fasted	1.06 ± 0.05	0.82 ± 0.08∗
Blood glucose [mg/dl]	*ad libitum* fed	154.22 ± 11.75	156.82 ± 6.72
	fasted	133 ± 8	98 ± 7∗∗
Insulin [ng/ml]	*ad libitum* fed	2.37 ± 0.25	1.02 ± 0.07∗∗∗
	fasted	0.44 ± 0.05	0.37 ± 0.06

Blood was collected from *ad libitum*–fed and 16 h–fasted Plin5-Tg and Wt mice (24w, n = 5–11). Data are presented as means ± SEM. Statistical significance was determined by unpaired Student's *t* test (∗*p* < 0.05; ∗∗*p* < 0.01; ∗∗∗*p* < 0.001 for effect of genotype).

**Figure 4:**
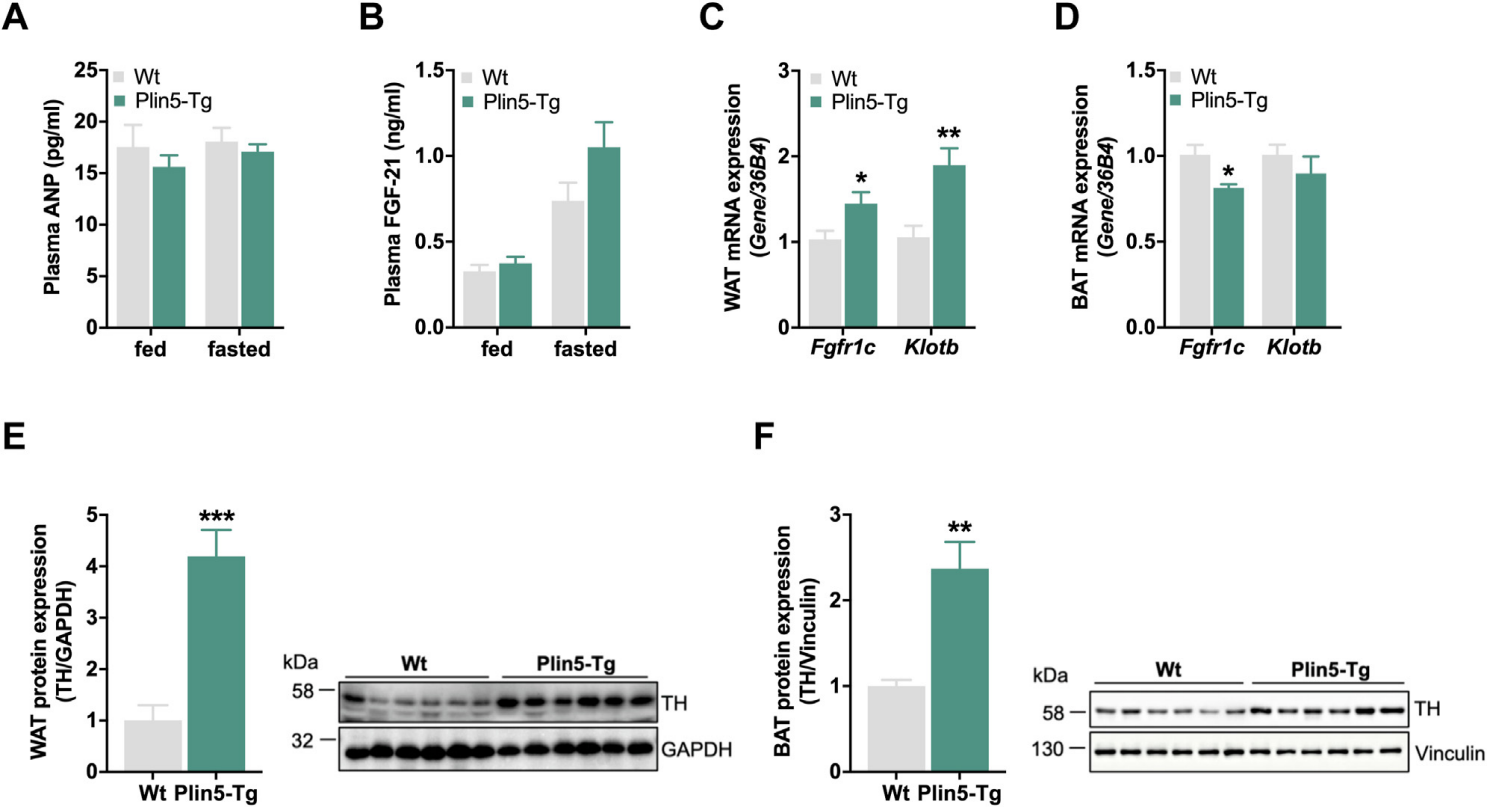
**Increased sympathetic innervation for ß-adrenergic stimulation.***A*, plasma ANP levels (15 weeks, *ad libitum* fed, n = 9–11) and *B*, plasma FGF21 levels under *ad libitum*–fed and 16 h–fasted conditions (28 weeks, n = 6–9). *C*, WAT (perigonadal AT) and (*D*) BAT mRNA expression of *Fgfr1c* and *Klotb* relative to *36B4* reference gene determined by qPCR with WT mice arbitrarily set to 1 for each gene. (26 weeks, *ad libitum* fed, n = 6). Protein expression of TH in (*E*), WAT (perigonadal AT) and (*F*), BAT. *Left*: quantification relative to GAPDH or Vinculin (26–30 weeks, *ad libitum* fed, n = 5–6). *Right*: representative immunoblots. Data are presented as means ± SEM. Statistical significance was determined using unpaired Student's *t* test (∗*p* < 0.05; ∗∗*p* < 0.01; ∗∗∗*p* < 0.001 for effect of genotype). BAT, brown adipose tissue; *Klotb*, ß-Klotho; qPCR, quantitative PCR; TH, tyrosine hydroxylase; WAT, white adipose tissue.

Since mRNA expression of natriuretic peptides (*Anp* and *Bnp*, respectively) was increased in cardiac muscle of Plin5-Tg mice (Fig. 2*A*) and it has been shown that ANP and BNP can be released from the heart to stimulate adipocyte lipolysis and thermogenesis (19, 20, 21), we determined plasma ANP levels. In contrast to increased cardiac mRNA expression, plasma ANP levels were comparable between Plin5-Tg and WT mice (Fig. 4*A*), suggesting that systemic natriuretic peptide signaling was not responsible for induced adipocyte lipolysis in transgenic mice. Likewise, FGF21 has been demonstrated to act on adipose tissue lipolysis (22). Increased cardiac *Fgf21* expression (Fig. 2*A*) did not significantly impact plasma FGF21 levels in Plin5-Tg mice (Fig. 4*B*). Yet, mRNA expression of the ß-Klotho–FGF receptor 1c complex, which is required for FGF21 signaling, was upregulated in WAT but not BAT (Fig. 4, *C* and *D*). Considering that adipose tissue lipolysis is mainly stimulated by catecholamines, we hypothesized that increased ß-adrenergic activation and consequently stimulation of PKA activity increases adipose tissue lipolysis in Plin5-Tg mice. In accordance, increased HSL phosphorylation at S563 (Fig. 3*D*), an established PKA site known to stimulate HSL activity, suggested increased ß-adrenergic activation in adipose tissue of Plin5-Tg mice. Tyrosine hydroxylase (TH), which is present in peripheral sympathetic neurons, is the rate-limiting enzyme synthesizing catecholamines, thereby modulating ß-adrenergic signaling. In agreement, TH protein expression was markedly increased in WAT (4.2-fold) and BAT (2.4-fold) of Plin5-Tg mice (Fig. 4, *E* and *F*), suggesting an upregulation of the catecholamine-mediated ß-adrenergic signaling pathway. Thus, elevated sympathetic innervation of adipose tissue most likely counteracts diet-induced adiposity in Plin5-Tg mice *via* increased adipocyte lipolysis and thermogenic activity.

### Plin5-Tg mice are protected from diet-induced glucose intolerance and insulin resistance

**Figure 5:**
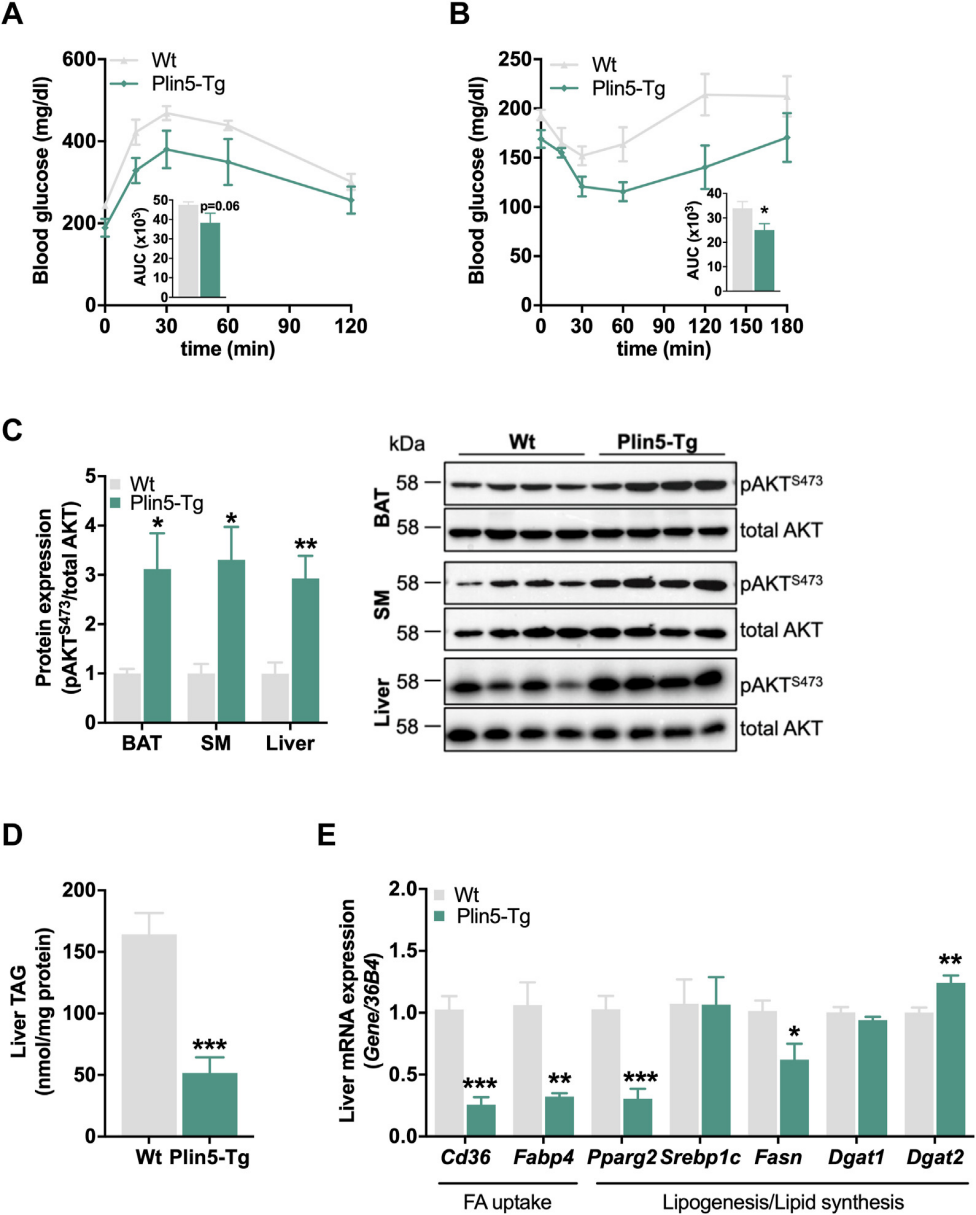
**Improved glucose tolerance and insulin signaling in Plin5-Tg micefed HFD.***A*, glucose tolerance test (GTT) of 6 h–fasted mice using 1.6 g glucose/kg body weight (28 weeks, n = 5–7). *B*, insulin tolerance test (ITT) of 4 h–fasted mice using 0.75 U insulin/kg body weight (30 weeks, n = 5–7). *C*, insulin signaling in the liver, BAT, and SM (quadriceps). Mice were injected with insulin at 0.75 U insulin/kg body weight. *Left*: quantification of phospho-AKT^S473^ relative to total AKT (20w, 16 h–fasted, n = 4). *Right*: representative immunoblots. *D*, hepatic triacylglycerol (TAG) levels (30 weeks, *ad libitum* fed, n = 5). *E*, liver mRNA expression of genes involved in FA uptake, lipogenesis, and lipid synthesis relative to *36B4* reference gene determined by qPCR with WT mice arbitrarily set to 1 for each gene (30 weeks, *ad libitum* fed, n = 5). Data are presented as means ± SEM. Statistical significance was determined using unpaired Student's *t* test (∗*p* < 0.05; ∗∗*p* < 0.01; ∗∗∗*p* < 0.001 for effect of genotype). BAT, brown adipose tissue; FA, fatty acid; HFD, high-fat diet; qPCR, quantitative PCR; SM, skeletal muscle.

Next, we assessed the impact of HFD on systemic glucose homeostasis and tissue-specific insulin signaling in Plin5-Tg mice. Blood glucose levels were reduced in short-term (0.8-fold; time 0 of glucose tolerance test/insulin tolerance test; Fig. 5, *A* and *B*) and overnight fasted (0.7-fold) HFD-fed Plin5-Tg mice accompanied by lower *ad libitum*–fed insulin levels (0.4-fold) (Table 1). Glucose clearance following a glucose (Fig. 5*A*) or insulin challenge (Fig. 5*B*) was increased in Plin5-Tg mice, suggesting improved glucose tolerance and insulin sensitivity in transgenic mice. Despite reduced adipose tissue mass, analysis of tissue-specific insulin signaling revealed unchanged phosphorylation of AKT S473 in WAT (Fig. S2*E*) but a drastic increase in insulin-stimulated AKT S473 phosphorylation in the BAT, skeletal muscle, and liver of Plin5-Tg mice (Fig. 5*C*). In the liver, augmented AKT S473 phosphorylation in Plin5-Tg mice was associated with lower TAG levels (Fig. 5*D*). Accordingly, hepatic mRNA expression of genes involved in FA uptake (*Cd36, Fabp4*) and lipogenesis (*Pparg2, Fasn*) was reduced (Fig. 5*E*). Thus, impaired cardiac TAG catabolism interferes with whole body energy and glucose homeostasis in Plin5-Tg mice–fed HFD.

### Increased BAT and WAT metabolic activity in Plin5-Tg mice fed HFD to maintain body temperature upon cold exposure

**Figure 6:**
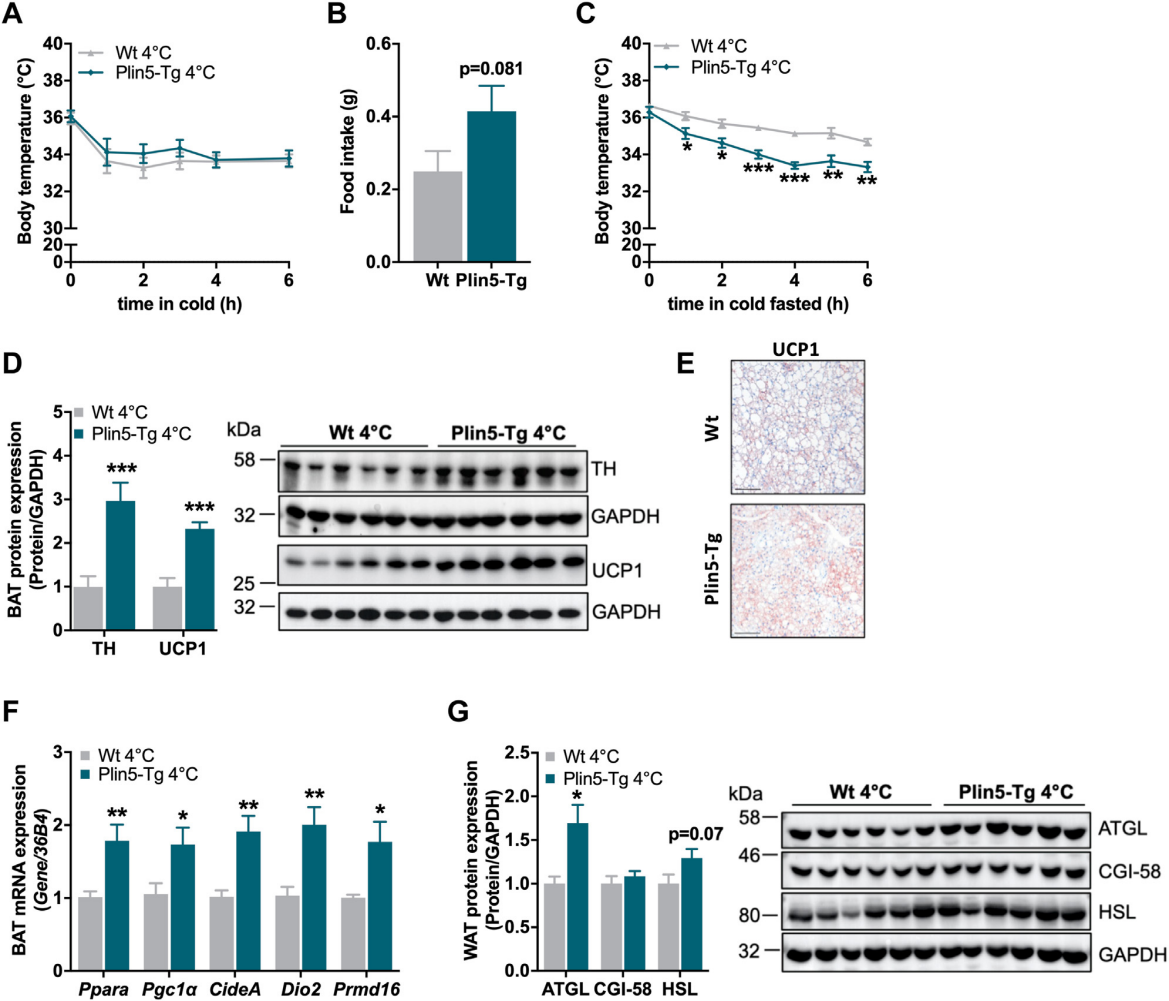
**Increased thermogenesis and lipolysis upon cold exposure in Plin5-Tg micefed HFD.***A*, body temperature and (*B*) food intake during 6 h cold exposure at 4 °C (30 weeks, n = 5–6). *C*, body temperature in fasted mice during 6 h cold exposure at 4 °C (30 weeks, n = 5). *D*, BAT protein expression of TH and UCP1. *Left*: quantification relative to GAPDH (30 weeks, 6 h fasted, n = 6). *Right*: representative immunoblots. *E*, representative images of UCP1 immunostaining in BAT. The scale bar represents 100 μm (30 weeks, 6 h fasted). *F*, BAT mRNA expression of genes involved in thermogenesis relative to *36B4* reference gene by qPCR with WT mice arbitrarily set to 1 for each gene (30 weeks, 6 h fasted, n = 6). *G*, WAT (perigonadal AT) protein expression of lipolytic proteins. *Left*: quantification of ATGL, CGI-58, and HSL relative to GAPDH (30 weeks, 6 h fasted, n = 6). *Right*: representative immunoblots. Data are presented as means ± SEM. Statistical significance was determined using unpaired Student's *t* test (∗*p* < 0.05; ∗∗*p* < 0.01; ∗∗∗*p* < 0.001 for effect of genotype). ATGL, adipose triglyceride lipase; BAT, brown adipose tissue; CGI, comparative gene identification; HFD, high-fat diet; HSL, hormone-sensitive lipase; qPCR, quantitative PCR; TH, tyrosine hydroxylase; UCP1, uncoupling protein 1; WAT, white adipose tissue.

Reduced BAT mass (Fig. 3*C*) and increased UCP1 expression (Fig. 3, *H* and *I*) in Plin5-Tg at room temperature indicated augmented thermogenic BAT activity. We were wondering whether Plin5-Tg mice could cope with acute cold exposure as an additional metabolic stress factor. Body temperature during an acute 6 h cold exposure with free access to food was comparable between Plin5-Tg and WT mice (Fig. 6*A*) albeit transgenic mice exhibited a trend toward increased food consumption (Fig. 6*B*). Food deprivation moderately reduced body temperature of Plin5-Tg mice in response to acute cold exposure (Fig. 6*C*), suggesting that Plin5-Tg mice need more energy intake to maintain body temperature. The significant increase in TH and UCP1 protein expression in BAT (Fig. 6, *D* and *E*) together with increased mRNA expression of genes involved in thermogenesis including *Ppara*, *Pgc1a*, *Dio2*, and *Prmd16* (Fig. 6*F*) corroborated increased BAT thermogenic activity. Furthermore, significantly elevated *lipoprotein lipase* (LPL) protein levels in the BAT of Plin5-Tg mice (Fig. S3*A*) indicated increased exogenous lipid requirement. In line, plasma TAG and FA but not total cholesterol levels were higher in 6 h–fasted Plin5-Tg mice than WT mice upon cold exposure (Fig. S3, *B–D*) and may indicate increased lipid flux to BAT. In contrast, blood glucose and insulin levels were reduced in Plin5-Tg mice in the fed and fasted state in response to cold stress (Fig. S3, *E* and *F*). In line with increased plasma FA levels, TH (Fig. S3*G*) as well as ATGL and HSL protein levels were increased in the WAT of transgenic mice (Fig. 6*G*), indicating augmented adipocyte lipolysis. Together, Plin5-Tg mice upregulate lipolytic and thermogenic activity in adipose tissue in response to acute cold stress at 4 °C and most likely at room temperature as an attempt to maintain body temperature, thereby counteracting diet-induced obesity.

### Thermoneutrality abrogates resistance toward HFD-induced obesity of Plin5-Tg mice

**Figure 7:**
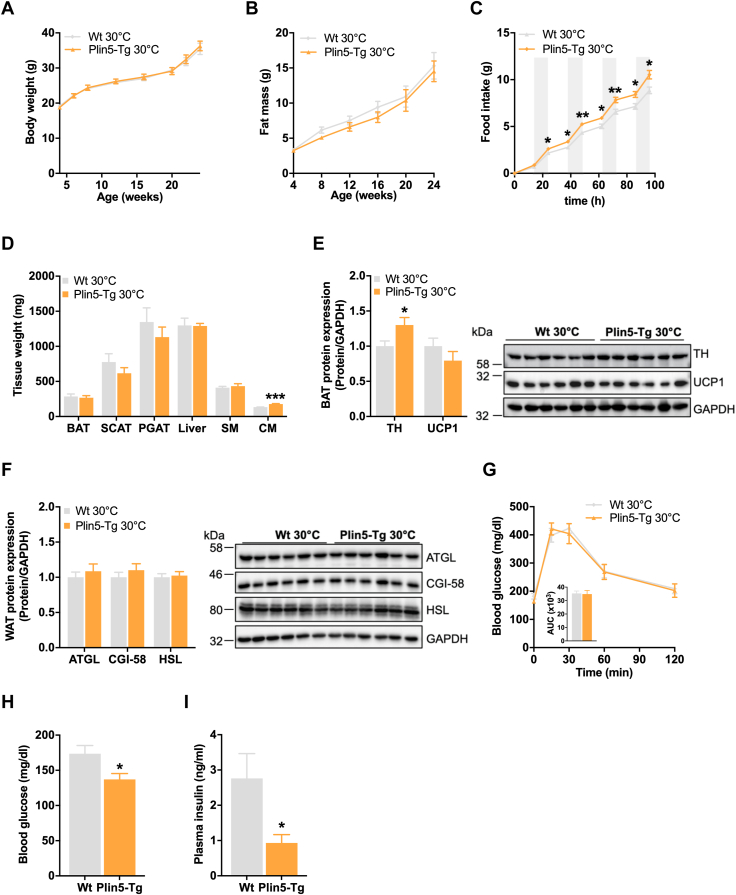
**Normal weight gain of HFD-fed Plin5-Tg mice at thermoneutrality.***A*, longitudinal body weight and (*B*) total fat mass at 30 °C (4–26 weeks, *ad libitum* fed, n = 6–7). *C*, cumulative food intake (14 weeks, *ad libitum* fed, n = 7). *D*, tissue weights (26 weeks, *ad libitum* fed, n = 6–7). *E*, protein expression of TH and UCP1 in BAT. *Left*: quantification relative to GAPDH (26 weeks, *ad libitum* fed, n = 6). *Right*: representative immunoblots. *F*, protein expression of lipolytic proteins in WAT (perigonadal AT). *Left*: quantification of ATGL, CGI-58, and HSL relative to GAPDH (26 weeks, *ad libitum* fed, n = 6). *Right*: representative immunoblots. *G*, glucose tolerance test (GTT) of 6 h–fasted mice using 1.6 g glucose/kg body weight (17 weeks, n = 7). *H*, blood glucose and (*I*) plasma insulin levels in *ad libitum*–fed state (26 weeks, n = 5–7). Data are presented as means ± SEM. Statistical significance was determined by unpaired Student’s *t* test (∗*p* < 0.05; ∗∗*p* < 0.01; ∗∗∗*p* < 0.001 for effect of genotype). ATGL, adipose triglyceride lipase; BAT, brown adipose tissue; CGI, comparative gene identification; HFD, high-fat diet; HSL, hormone-sensitive lipase; TH, tyrosine hydroxylase; UCP1, uncoupling protein 1; WAT, white adipose tissue.

To investigate whether an increased energy demand to maintain body temperature protects Plin5-Tg mice from diet-induced obesity, we next assessed the impact of HFD on systemic energy homeostasis at thermoneutrality. In contrast to mice housed at room temperature, body weight (Fig. 7*A*) and total fat mass (Fig. 7*B*) were comparable between Plin5-Tg and WT mice at thermoneutrality despite increased cumulative food intake of Plin5-Tg mice (Fig. 7*C*). Except for the expected increase in cardiac muscle mass of transgenic mice, tissue weights of adipose tissue depots, the liver, and the skeletal muscle were comparable to WT (Fig. 7*D*). In agreement with unchanged adipose tissue mass, UCP1 protein expression in BAT (Fig. 7*E*) as well as ATGL, CGI-58, and HSL protein expression in WAT (Fig. 7*F*) were similar among Plin5-Tg and controls. Likewise, glucose clearance following a glucose challenge was comparable between genotypes (Fig. 7*G*). Nonetheless, *ad libitum*–fed blood glucose (Fig. 7*H*) and plasma insulin levels (Fig. 7*I*) were still reduced in transgenic mice, indicating sustained insulin sensitivity upon diet-induced obesity. Together, Plin5-Tg mice counteract diet-induced obesity *via* elevated lipolytic and thermogenic activity in adipose tissue at room temperature (and upon cold exposure) but not at thermoneutrality. In contrast, improved insulin sensitivity is independent of ambient temperature, which is most likely a consequence of altered cardiac energy metabolism, that is, low cardiac lipolysis and likely increased glucose utilization in Plin5-Tg mice.

## Discussion

The global prevalence of obesity steadily increases and is associated with a rise in cardiovascular disease as the leading cause of death in the industrialized world (8). Obese individuals typically show an increase in cardiac TAG levels and LV mass eventually progressing to heart failure (1). We have recently demonstrated in Plin5-Tg mice that cardiac TAG accumulation and reduced utilization of FAs as energy substrate are compatible with normal heart function due to physiologic eccentric cardiac growth (18). The protection from lipotoxic heart disease in Plin5-Tg mice prompted us to study heart function and energy metabolism under HFD-induced metabolic stress. In strong contrast to chow diet, HFD provokes pathological LV remodeling and increased cardiac fibrosis despite preserved ejection fraction in 4-month-old mice. However, declined heart function in 1-year-old mice indicates that restricted cardiac lipolysis and the inability to increase FA utilization in Plin5-Tg mice trigger the development of lipotoxic heart dysfunction with age. The failure to increase LV ejection fraction upon exercise in individuals with increased LV mass (23) suggests that exceeding a certain threshold of LV mass impairs heart function. In line, our data on heart mass and function suggest that the limited capacity of (eccentric) heart growth and myocardial FA oxidation may provoke adverse cardiac remodeling in Plin5-Tg mice on HFD.

Cardiomyopathy development and heart failure caused by augmented ß-adrenergic signaling in response to myocardial damage is an adaptation to maintain cardiac output (24). However, sustained and excess ß-adrenergic stimulation are detrimental to heart physiology and may also increase adipose tissue lipolysis thereby further accelerating cardiac FA flux and damage. In accordance with this assumption, lowering adipose tissue lipolysis *via* deletion or pharmacological inhibition of ATGL ameliorated cardiac dysfunction in animal models with heart failure (5, 6, 7). In these studies, cardiomyopathy and heart failure were induced by aortic banding or transverse aortic construction where cardiomyopathy develops in a relative short time. However, the impact of sustained cardiomyopathy and metabolic stress on whole body energy homeostasis is less established. Here we show that prolonged cardiomyopathy in Plin5-Tg mice strongly interferes with whole body energy homeostasis thereby counteracting HFD-induced obesity and maintaining glucose tolerance. These metabolic changes are not the consequence of heart failure as ejection fraction was similar in 4-month-old mice with HFD feeding starting at 4 to 6 weeks of age. Augmented cardiac glucose utilization is compatible with normal heart function in GLUT1 transgenic mice–fed a chow diet and protects toward contractile dysfunction after chronic pressure overload (25). In contrast to Plin5-Tg mice, GLUT1 transgenic mice are obese when fed HFD and develop lipotoxic heart dysfunction (26). Accordingly, low cardiac lipolysis in Plin5-Tg mice divergently impacts heart function and whole body energy homeostasis compared to GLUT1 transgenic mice and further implicates that the metabolic changes in Plin5-Tg mice are not the consequence of heart failure. Interestingly, Sun *et al.* (27) showed that postnatal deletion of histone deacetylase 3 is compatible with normal life span. The mice show mild cardiac hypertrophy on chow diet, but switching to HFD provokes lethal heart dysfunction already starting after 3 weeks on HFD. The authors discussed that mitochondrial dysfunction due to increased cardiac FA uptake provokes deleterious lipotoxic cardiomyopathy similar to mice with cardiac-specific overexpression of PPARα (4). Our data indicate that FA uptake is reduced in Plin5-Tg mice and may counteract the development of lipotoxic heart dysfunction on HFD. In line, impaired lipolysis in WAT either *via* loss of ATGL (28) or HSL (29) downregulates FA uptake and lipogenesis in WAT likely *via* reduced generation of ligands for nuclear transcription factors. It is conceivable that low cardiac lipolysis in Plin5-Tg mice (16) and reduced PPARα-activated gene expression cause a similar adaptation and lower cardiac FA uptake, thereby protecting mice from the development of lethal cardiomyopathy.

Interestingly, cardiac-specific overexpression of MED13, a subunit of the mediator complex involved in nuclear hormone receptor–mediated gene transcription, protects mice from HFD-induced obesity and insulin resistance (30, 31). MED13 transgenic mice show reduced expression of metabolic genes in the heart, whereas metabolic activity increases in the adipose tissue and liver to counteract obesity development. However, this study did not elucidate which cardiometabolic changes and/or endocrine/paracrine factors may stimulate whole body energy metabolism. Similar to Plin5-Tg mice, plasma ANP levels were unchanged in MED13 transgenic mice despite significantly increased cardiac *Anp* expression. Reduced HFD-induced obesity in Plin5-Tg mice is most likely the consequence of increased ß-adrenergic signaling and lipolysis in adipose tissue and stimulation of BAT thermogenesis triggered by impaired cardiac FA oxidation and cardiomyopathy. In accordance, ATGL expression and TAG hydrolytic activity were significantly increased in WAT (both, perigonadal and subcutaneous adipose tissue [*data not shown*]), which substantially contributes to adipose tissue lipolysis and FA supply into the circulation (32). The activation of ß-adrenergic receptors and the concomitant rise in cellular cAMP levels activates PKA, which phosphorylates PLIN1 and HSL to increase lipolysis (33). The significant rise in phosphorylated (S563) HSL levels further suggested increased ß-adrenergic signaling and lipolysis in WAT. Interestingly, adipose tissue–specific ATGL overexpression also protects mice from HFD-induced obesity and insulin resistance without increasing circulating FA levels due to augmented adipose tissue FA oxidation (34).

Our data suggest that stimulation of adipose tissue lipolysis and BAT thermogenesis plays a central role in lipid and energy homeostasis of Plin5-Tg mice on HFD. Accordingly, TH and UCP1 as well as lipolytic protein levels were markedly increased in the BAT of Plin5-Tg mice at room temperature. The important role of BAT in obesity resistance of Plin5-Tg mice is further supported by the strongly increased expression of UCP1 and TH upon acute cold exposure, whereas UCP1 expression was unchanged at thermoneutrality. A recent study demonstrates the induction of insulin-mediated lipid uptake into BAT upon acute cold exposure or ß_3_-adrenergic activation, which is important for TAG replenishment and adaptive thermogenesis (35). In this study, acute cold exposure increased plasma TAG levels. Furthermore, the study by Chondronikola *et al.* (36) showed that cold exposure increases the expression of CD36 and LPL in human BAT. The significant increase in LPL protein levels in the BAT of Plin5-Tg mice upon acute cold exposure together with an increase in plasma TAG and FA levels upon moderate fasting suggest a similar adaptation in Plin5-Tg mice upon acute cold exposure and that ß-adrenergic stimulation of adipocyte lipolysis channels FA into BAT as thermogenic fuel. The similar phenotype of WT and Plin5-Tg mice at thermoneutrality further indicated that increased BAT thermogenesis counteracts obesity development even at room temperature. Studies in rodents demonstrate that BAT activity is also stimulated by the intake of a carbohydrate-enriched meal typically referred to as diet-induced thermogenesis (37, 38). However, the role of BAT in diet-induced thermogenesis is a matter of dispute and controversially discussed (39, 40, 41). Our data show that the diet has a profound impact on BAT activity as feeding a chow diet does not alter body weight and BAT mass of Plin5-Tg mice in contrast to mice kept on HFD.

In summary, this study reveals novel insights into the metabolic crosstalk between the heart and peripheral tissues including BAT, WAT, and the liver. Restricted cardiac FA catabolism under conditions of increased dietary fat intake in Plin5-Tg mice impacts whole body energy metabolism *via* the stimulation of WAT and BAT activity and the reduction of hepatic lipid uptake. Lowering cardiac lipolysis or FA oxidation to protect cardiomyocytes from reperfusion injury and cardiac damage (8) can be a therapeutic strategy to ameliorate cardiac lipotoxicity and may increase cardiac efficiency if diets are carefully controlled.

## Experimental procedures

### Animals

Mice with cardiomyocyte-specific PLIN5 overexpression (16) were housed in a specific pathogen-free facility under standard conditions (21–23 °C, 14:10 h light:dark cycle) with *ad libitum* access to water and a standard laboratory chow diet (11 kcal% fat; ssniff Spezialitäten GmbH) or HFD (45 kcal% fat; ssniff Spezialitäten GmbH). HFD was initiated after weaning at 4 to 6 weeks of age. For acute cold studies, mice were single-housed and transferred to 4 °C for 6 h with *ad libitum* access to water and food or fasted for 6 h. For studies at thermoneutrality, mice were transferred to a room with an ambient temperature of 30 °C and 35 to 50% humidity at the time of HFD initiation for 20 weeks. All animal experiments were approved by the Austrian Federal Ministry for Science and Research and by the ethics committee of the University of Graz and the University of Veterinary Medicine Vienna (protocol number BMWFW-66.007/0006-WF/V/3 b/2014) according to the EU (Directive 2010/63/EU) ethical guidelines. For all studies, male mice at the age of 15 to 30 weeks were used. Experiments were performed in the *ad libitum*–fed state on HFD if not otherwise indicated.

### Metabolic phenotyping

Body composition was determined by NMR spectroscopy using a TD-NMR miniSpec Live Mice Analyzer (Bruker Optics). For metabolic phenotyping, mice were single-housed in a laboratory animal monitoring system (PhenoMaster, TSE Systems) with *ad libitum* access to HFD and water. Food intake, locomotor activity, and oxygen consumption rate were determined for light and dark phase on four consecutive days. Body temperature was measured using a rectal probe RET-3 (Physitemp). For glucose tolerance test, mice were injected intraperitoneally (ip) with 1.6 g per kg body weight following a 6 h fast. For insulin tolerance test, mice were injected ip with 0.75 IU insulin per kg body weight following a 4 h fast. Blood glucose was determined by Wellion CALLA glucometer (Med Trust). Plasma lipid parameters were determined using following commercial kits: FA (NEFA-HR2, Wako Diagnostics), TAG (Infinity triglycerides, Thermo Fisher Scientific), glycerol (free glycerol reagent, Sigma-Aldrich), total cholesterol (cholesterol CHOD-PAP kit; Roche Applied Science), and ketone bodies (β-Hydroxybutyrate Assay Kit, Cayman Chemical). Plasma insulin levels were determined using Ultra-Sensitive Mouse Insulin ELISA Kit (Crystal Chem). Plasma ANP levels were determined using Atrial Natriuretic Peptide EIA Kit (Sigma-Aldrich). Plasma FGF21 levels were determined using Rat/Mouse FGF21 ELISA Kit (Sigma-Aldrich).

### Magnetic resonance imaging

MRI was performed on a 7T small animal MRI platform (Bruker BioSpec) equipped with a 660 mT/m gradient system. A cryogenic cooled transmit/receive unit was used to gain high resolution cine MR images. Mice were anesthetized with 1.5 to 2.0% isoflurane in O_2_ (1 L/min) through a nose cone. Body temperature was maintained by a water-heated animal bed. Short-axis views of the hearts were planned on ECG-triggered long-axis cine images. Additional shimming was carried out with an ECG-triggered voxel-based shimming procedure covering the epicardium of the left ventricle at end-diastole. A true short-axis ECG and respiration-gated cine gradient–recalled echo sequence followed. Measurement parameters included the following: pulse repetition time/echo time = 5.4/1.9 ms, α = 50°, two averages, an image matrix of 256 × 256 at a field of view of 25 × 25 mm^2^. Eight consecutive slices of 1 mm thickness covered the heart from apex to base. Eighteen cardiac frames were recorded. Triggered total imaging time was approximately 3 min per slice. The myocardial LV wall was manually segmented in all short-axis views using itk-SNAP, version 3.6.0 (42) to obtain LV epicardial and endocardial volumes, while papillary muscles were excluded from the lumen. LV mass was calculated from myocardial volume using a tissue density of 1.04 g/cm³. Ejection fraction was obtained by the difference of end-diastolic and end-systolic endocardial volume divided by the end-diastolic endocardial volume multiplied by 100%. LV septum (LV_S), posterior wall (LV_PW) thickness, and LV endocardial diameter (LV_DIA) were measured manually in the mid-axial slice using ImageJ. Relative wall thickness was calculated as RWT = (LV_S + LV_PW)/LV_DIA ∗100.

### Gene and protein expression

For gene expression, total RNA was extracted *via* TRIzol reagent (Thermo Fisher Scientific) and treated with DNaseI (New England Biolabs). Complementary DNA synthesis was performed using the LunaScript RT SuperMix Kit (New England Biolabs). Reverse transcription-PCR was performed as previously described (16). Relative mRNA levels were quantified according to the ΔΔCT method with *36b4* as the reference gene. Primer sequences are listed in Table S1.

For protein expression, snap-frozen tissue was homogenized in ice-cold buffer A (0.25 M sucrose, 1 mM EDTA, 1 mM DTT, 20 μg/ml leupeptin, 2 μg/ml antipain, 1 μg/ml pepstatin, and phosphatase inhibitor PhosStop [Roche]; pH 7) using Ultra-Turrax homogenizer and centrifuged for 15 min at 1000*g* and 4 °C. Infranatant was collected and protein concentration was determined with Bio-Rad Protein Assay reagent (Bio-Rad Laboratories). Western blot analysis was performed according to standard protocols. Antibodies are listed in Table S2.

### Insulin signaling in tissue

For tissue-specific insulin signaling, 16 h–fasted mice were injected ip with saline or 0.75 IU insulin per kg body weight and sacrificed after 10 min. Snap-frozen tissues were homogenized in ice-cold lysis buffer (50 mM Tris–HCl, 150 mM NaCl, 1 mM EDTA, 1% NP-40, 20 μg/ml leupeptin, 2 μg/ml antipain, 1 μg/ml pepstatin, and phosphatase inhibitor PhosStop [Roche]; pH 7.5) using Ultra-Turrax Homogenizer (IKA). After centrifugation for 30 min at 20,000*g* and 4 °C, infranatant was collected. Protein concentrations were determined using Pierce BCA protein assay kit.

### Histology

Tissues were fixed in 4% buffered formaldehyde, embedded in paraffin, and 2 μm sections were prepared using a Microm HM 430 (Zeiss). Sections were stained with H&E or Sirius Red using standard protocols. UCP1 immunohistochemistry was performed according to standard protocols *via* Dako Real Envision Detection System using UCP1 antibody (1:50, ab10983, Abcam).

### Lipid extraction

Total tissue lipids were extracted according to the method of Folch (43). Snap-frozen samples were homogenized in 1 ml CHCl_3_:MeOH (2:1) using a Retsch mill. After adding 200 μl of water, samples were vortexed and centrifuged for 10 min at 5000×*g* and 4 °C. The organic phase was collected and the extraction was repeated by adding 500 μl of CHCl_3_. Organic phases were pooled and evaporated under nitrogen steam. Lipid extracts were resuspended in 0.1% Triton X-100 O/N at 40 °C and 1000 rpm. TAG and total cholesterol content were determined using following commercial kits: TAG (Infinity triglycerides; Thermo Fisher Scientific) and total cholesterol (cholesterol CHOD-PAP kit; Roche Applied Science). Protein pellets were air dried O/N at 60 °C and dissolved in 0.3 M NaOH containing 0.1% SDS at 60 °C and 600 rpm for 4 to 6 h. Protein concentrations were determined using Pierce BCA protein assay kit (Thermo Fisher Scientific) according to manufacturer’s instructions using bovine serum albumin as a standard.

### TAG hydrolase activity

TAG hydrolase activity assay was performed as previously described (29). TAG substrate contained 300 μM triolein, 10 μCi per ml [^3^H] triolein, and 45 μM PC/PI (3/1, M/M). Lipids were dried under N_2_ and emulsified by sonication in 100 mM K-phosphate buffer (pH 7.4) followed by the addition of 5% FA-free bovine serum albumin. Twenty micrograms adipose tissue lysate in 100 μl buffer A was incubated with 100 μl substrate for 1 h at 37  °C. Reactions were terminated by the addition of 3.25 ml methanol/chloroform/*n*-heptane (10/9/7, v/v/v) and 1 ml 100 mM K-carbonate (pH 10.5). Then, samples were vigorously vortexed and centrifuged at 2000*g* for 10 min. The radioactivity in the upper phase was determined by liquid scintillation counting.

### Statistical analysis

Data are presented as means ± SEM. Statistical significance was determined by unpaired Student’s two-tailed *t* test. *p* values of < 0.05 were considered statistically significant. The exact numbers of replicates are presented in individual figures.

## Data availability

The authors confirm that the data supporting the findings of this study are available within the article. Primary data are available from the corresponding authors upon reasonable request.

## Supporting information

This article contains supporting information.

## Conflict of interest

The authors declare that they have no conflicts of interest with the contents of this article.
