# Supplementary material for: Adverse cardiac remodeling augments adipose tissue ß-adrenergic signaling and lipolysis counteracting diet-induced obesity
Source: J Biol Chem. 2023 May 5;299(6):104788. doi: 10.1016/j.jbc.2023.104788 (PMC10318461; doi:10.1016/j.jbc.2023.104788)
Supplement: Supporting information [file mmc1.docx]

**Supporting information**

**Adverse cardiac remodeling augments adipose tissue ß-adrenergic signaling and lipolysis counteracting diet-induced obesity**

Stephanie Kolleritsch^1^, Laura Pajed^1^, Anna Tilp^1^, Victoria Hois^2^, Isabella Potoschnig^1^, Benedikt Kien^1^, Clemens Diwoky^1^, Gerald Hoefler^3,4^, Gabriele Schoiswohl^1,4,5*^, and Guenter Haemmerle^1*^

**Supplemental Table 1: Primer sequences for RT-PCR.**

| **Gene name** | **Primer** | **Sequence** |
| --- | --- | --- |
| 36B4 | *36b4-fw* | 5’- GCTTCATTGTGGGAGCAGACA -3’ |
|  | *36b4-rv* | 5’- CATGGTGTTCTTGCCCATCAG -3’ |
| Acyl-CoA oxidase 1 | *Aox1-fw* | 5’- AGATTGGTAGAAATTGCTGCAAAA -3’ |
|  | *Aox1-rv* | 5’- ACGCCACTTCCTTGCTCTTC -3’ |
| Atrial natriuretic peptide | *Anp-fw* | 5‘- CTGCTTCGGGGGTAGGATTG -3‘ |
|  | *Anp-rv* | 5‘- TTCGGTACCGGAAGCTGT -3‘ |
| Beta klotho | *Klotb-fw* | 5’-TGTTCTGCTGCGAGCTGTTAC -3‘ |
|  | *Klotb-rev* | 5’-TTATCCCATATTGCTTTCCCGTC -3‘ |
| Brain natriuretic peptide | *Bnp-fw* | 5’- GCTGCTTTGGGCACAAGATAG -3’ |
|  | *Bnp-rv* | 5’- GCAGCCAGGAGGTCTTCCTA -3’ |
| Carnitine palmitoyltransferase 1B | *Cpt1b-fw* | 5’- CGAGGATTCTCTGGAACTGC-3’ |
|  | *Cpt1b-rv* | 5’- GGTCGCTTCTTCAAGGTCTG -3’ |
| Cell Death Inducing DFFA Like Effector A | *Cidea-fw* | 5’- TGCTCTTCTGTATCGCCCAGT -3’ |
|  | *Cidea-rv* | 5’- GCCGTGTTAAGGAATCTGCTG -3’ |
| Cluster of differentiation 11c | *Cd11c-fw* | 5’- CAGTGACCCCGATCACTCTT -3’ |
|  | *Cd11c-rv* | 5’- CACCACCAGGGTCTTCAAGT -3’ |
| Cluster of differentiation 36 | *Cd36-fw* | 5’- GAACCTATTGAAGGCTTACATCC-3’ |
|  | *Cd36-rv* | 5’- CCCAGTCACTTGTGTTTTGAAC-3’ |
| Diacylglycerol acyltransferase 1 | *Dgat1-fw* | 5’- GTGCCATCGTCTGCAAGATTC -3’ |
|  | *Dgat1-rv* | 5’- GCATCACCACACACCAATTCAG -3’ |
| Diacylglycerol acyltransferase 2 | *Dgat2-fw* | 5’- TTCCTGGCATAAGGCCCTATT -3’ |
|  | *Dgat2-rv* | 5’- AGTCTATGGTGTCTCGGTTGAC -3’ |
| Deiodinase, iodothyronine,  type II | *Dio2-fw* | 5’- CAGTGTGGTGCACGTCTCCAATC -3’ |
|  | *Dio2-rv* | 5’- TGAACCAAAGTTGACCACCAG -3’ |
| F4/80 | *F4/80-fw* | 5’- GGATGTACAGATGGGGGATG -3’ |
|  | *F4/80-rv* | 5’- CATAAGCTGGGCAAGTGGTA -3’ |
| Fatty acid binding protein 1 | *Fabp1-fw* | 5‘- gggaagaaaatcaaactcaccatc -3‘ |
|  | *Fabp1-rv* | 5‘- agttgtcaccattttattgtcacc -3‘ |
| Fatty acid binding protein 3 | *Fabp3-fw* | 5’- cccctcagctcagcaccat -3’ |
|  | *Fabp3-rv* | 5’- cagaaaaatcccaacccaagaat -3’ |
| Fatty acid binding protein 4 | *Fabp4-fw* | 5’- AAGGTGAAGAGCATCATAACCCT -3’ |
|  | *Fabp4-rv* | 5’- TCACGCCTTTCATAACACATTCC -3’ |
| Fatty acid synthase | *Fasn-fw* | 5’- TCCTGGAACGAGAACACGATCT -3’ |
|  | *Fasn-rv* | 5’- GAGACGTGTCACTCCTGGACTTG -3’ |
| Fibroblast growth factor 21 | *Fgf21-fw* | 5’- TCCAAATCCTGGGTGTCAAA -3’ |
|  | *Fgf21-rev* | 5’- CAGCAGCAGTTCTCTGAAGC -3’ |
| FGF receptor 1c | *Fgfr1c-fw* | 5’ GCCAGACAACTTGCCGTATG -3’ |
|  | *Fgfr1c-rv* | 5’ ATTTCCTTGTCGGTGGTATTAACTC -3’ |
| Glucose transporter 1 | *Glut1-fw* | 5′- GCCCCCAGAAGGTTATTGA -3′ |
|  | *Glut1-rv* | 5′- CGTGGTGAGTGTGGTGGATG -3′ |
| Glucose transporter 4 | *Glut4-fw* | 5’- GTGACTGGAACACTGGTCCTA -3’ |
|  | *Glut4-rv* | 5’- CCAGCCACGTTGCATTGTAG -3’ |
| Interleukin-6 | *Il-6-fw* | 5’- GAGGATACCACTCCCAACAGACC -3 |
|  | *Il-6-rv* | 5’- AAGTGCATCATCGTTGTTCATACA -3’ |
| Lipoprotein lipase | *Lpl-fw* | 5’-TCCAGCCAGGATGCAACA -3’ |
|  | *Lpl-rv* | 5’-CCACGTCTCCGAGTCCTCTCT-3’ |
| Long-chain acyl-CoA dehydrogenase | *Lcad-fw* | 5’- GGCAAAATACTGGGCATCTGA -3’ |
|  | *Lcad-rv* | 5’- CTCCGTGGAGTTGCACACAT -3’ |
| Medium-chain acyl-CoA dehydrogenase | *Macd-fw* | 5’- CAACACTCGAAAGCGGCTCA -3’ |
|  | *Macd-rv* | 5’- ACTTGCGGGCAGTTGCTTG -3’ |
| Myosin heavy chain 7 | *Myh7-fw* | 5’- GATGTTTTTGTGCCCGATGA -3’ |
|  | *Myh7-rv* | 5’- TGTCGAACTTGGGTGGGTT -3’ |
| Peroxisome proliferator-activated receptor alpha | *Ppara-fw* | 5’- GTACCACTACGGAGTTCACGCAT -3’ |
|  | *Ppara-rv* | 5’- CGCCGAAAGAAGCCCTTAC-3’ |
| Peroxisome proliferator-activated receptor gamma 2 | *Pparg2-fw* | 5’- CCAGAGCATGGTGCCTTCGCT -3’ |
|  | *Pparg2-rv* | 5’- CAGCAACCATTGGGTCAG -3’ |
| Peroxisome proliferator-activated receptor gamma coactivator 1-alpha | *Pgc1a-fw* | 5’- CCCTGCCATTGTTAAGACC -3’ |
|  | *Pgc1a-rv* | 5’- TGCTGCTGTTCCTGTTTTC -3’ |
| PR/SET Domain 16 | *Prmd16-fw* | 5’- CAGCACGGTGAAGCCATT C -3’ |
|  | *Prmd16-rv* | 5’- GCGTGCATCCGCTTGTG -3’ |
| Sterol response element binding protein 1c | *Srebp1c-fw* | 5’- GTTACTCGAGCCTGCCTTCAGG -3’ |
|  | *Srebp1c-rv* | 5’- CAAGCTTTGGACCTGGGTGTG -3’ |
| Very-long-chain acyl-CoA dehydrogenase | *Vlcad-fw* | 5’- ACCTTGCCAGGGCCTGAT -3’ |
|  | *Vlcad-rv* | 5’- TGGCCTGGTCACCGGTAA -3’ |

**Supplemental Table 2: Antibodies used for western blot analyses.**

| **Protein** | **Protein name** | **Company** | **Dilution used** |
| --- | --- | --- | --- |
| AKT | RAC-alpha serine/threonine-protein kinase | Cell Signaling, C67E7 | 1:1,000 in 5% BSA |
| pAKT^S473^ | RAC-alpha serine/threonine-protein kinase | Cell Signaling, 9271S | 1:1,000 in 5% BSA |
| ATGL | Adipose triglyceride lipase | Cell Signaling, 2138S | 1:1,000 in 5% milk |
| CGI-58 | Comparative gene identification-58 | Abnova, H00051099-M01 | 1:1,000 in 5% milk |
| GAPDH | Glyceraldehyde-3-phosphate dehydrogenase | Cell Signaling, 2118S | 1:20,000 in 5% milk |
| HSL | Hormone-sensitive lipase | Cell Signaling, 4107S | 1:3,000 in 5% milk |
| pHSL^S563^ | Hormone-sensitive lipase | Cell Signaling, 4139 | 1:1,000 in 5% BSA |
| LPL | Lipoprotein Lipase | kindly provided by *Stephan Young* | 1:500 in 5% milk |
| PLIN5 | Perilipin 5 | Progen, GP31 | 1:1,000 in 5% milk |
| TH | Tyrosine hydroxylase | Millipore, MAB318 | 1:1,000 in 5% milk |
| UCP1 | Uncoupling protein 1 | Abcam, ab10983 | 1:20,000 in 5% milk |
| Vinculin | Vinculin | Sigma-Aldrich, V9131 | 1:20,000 in 5% milk |

**
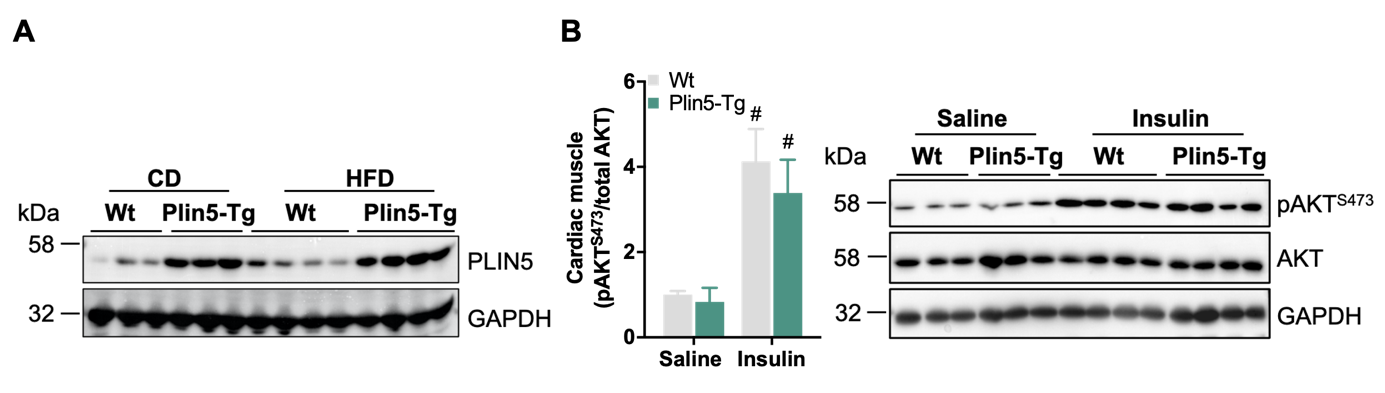
Supplemental Figure S1. Cardiac insulin signaling in Plin5-Tg mice fed HFD.** A, representative immunoblot of PLIN5 protein expression in CM of mice fed chow diet (CD) and HFD using GAPDH as loading control (30wk, *ad libitum* fed, n=3-4). B, insulin signaling in cardiac muscle. Mice were injected with saline or insulin at 0.75 U insulin/kg body weight. Left: Quantification of phospho-AKT^S473^ relative to total AKT (20wk, 16 h-fasted, n=3-4). Right: Representative immunoblots. Data are presented as means ± SEM. Statistical significance was determined using unpaired Student's t-test (^#^, p<0.05 for effect of treatment).

**
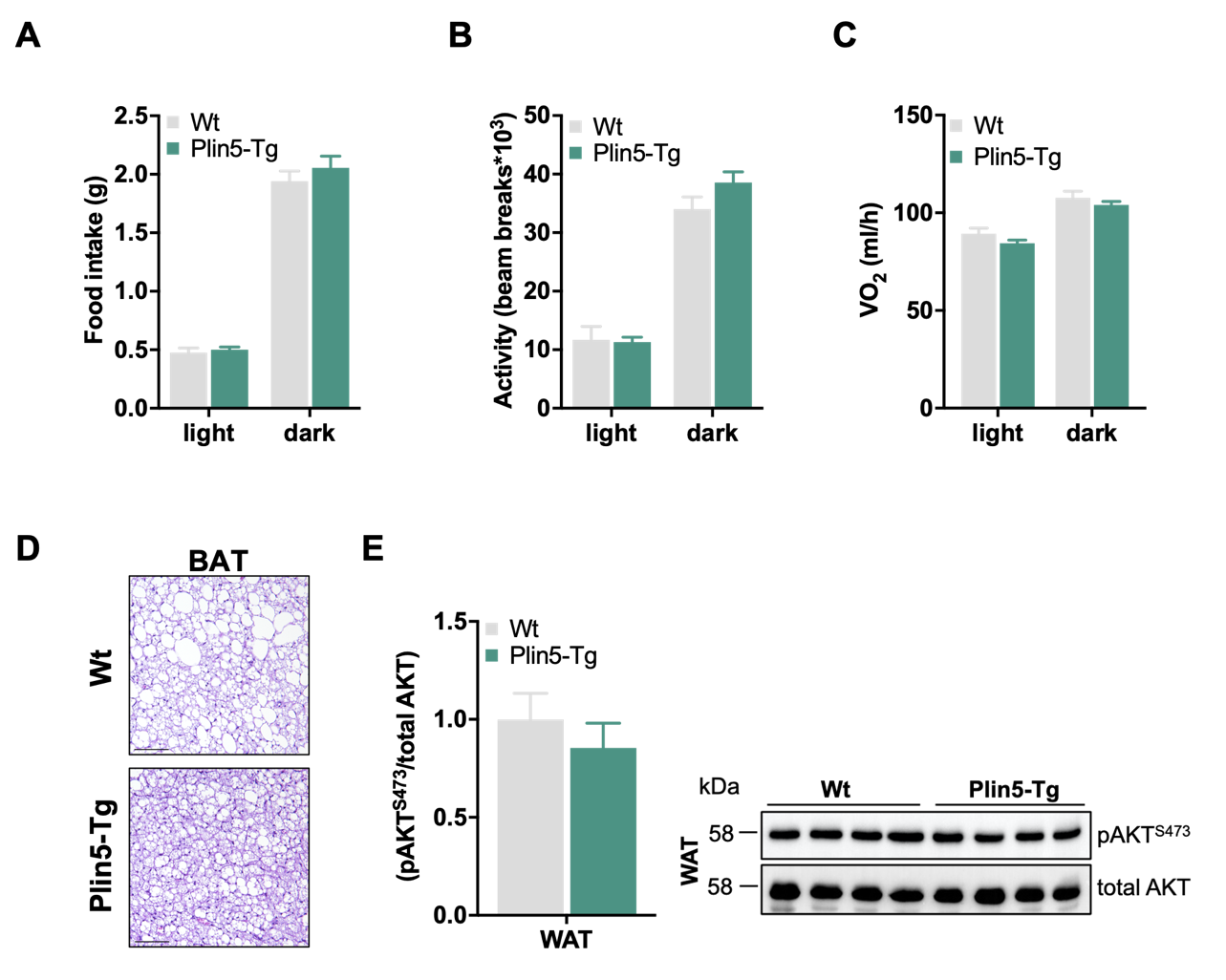
Supplemental Figure S2. Systemic energy metabolism in Plin5-Tg mice fed HFD.** Metabolic phenotyping of mice using a laboratory animal monitoring system: A, food intake, B, locomotor activity, and C, oxygen consumption rate was determined over 4 consecutive days at dark and light phase (17wk, *ad libitum* fed, n=6). D, representative histological images of BAT sections stained with hematoxylin and eosin. Scale bar: 100 µm (30wk, *ad libitum* fed). E, insulin signaling in WAT (perigonadal AT). Mice were injected with insulin at 0.75 U insulin/kg body weight. Left: Quantification of phospho-AKT^S473^ relative to total AKT (20w, 16 h-fasted, n=4). Right: Representative immunoblots. Data are presented as means ± SEM.

**
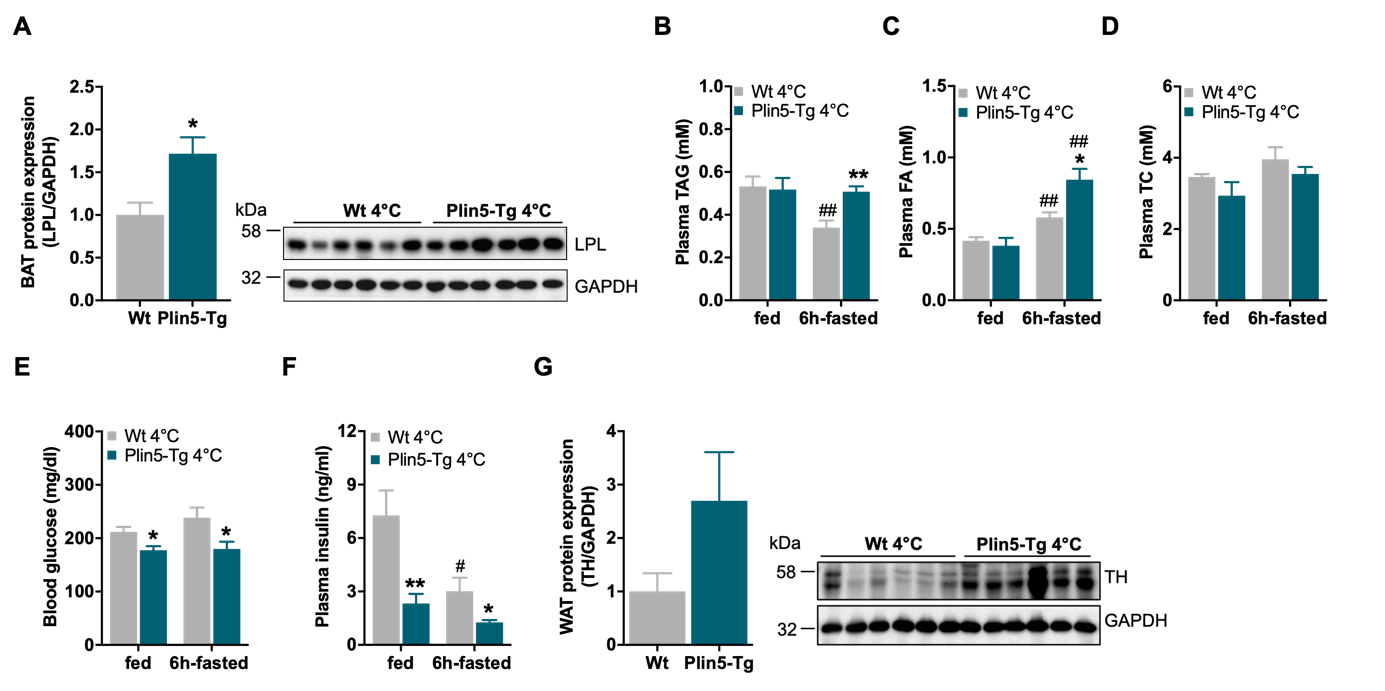
Supplemental Figure S3. Increased thermogenesis and lipolysis upon cold exposure in Plin5-Tg mice fed HFD.** A, protein expression of LPL in BAT. Left: Quantification relative to GAPDH (30wk, 6 h fasted, n=6). Right: Representative immunoblots. GAPDH immunoblot is the same as in Figure 6D. B, plasma TAG, C, FA, and D, total cholesterol (TC) levels in *ad libitum* fed and 6h-fasted mice following cold exposure at (30wks, n=5-6). E, blood glucose and F, plasma insulin levels in *ad libitum* fed and 6 h-fasted mice following cold exposure at 4°C (30wk, n=5-6). G, protein expression of TH in WAT (perigonadal AT). Left: Quantification relative to GAPDH (30wk, 6 h fasted, n=6). Right: Representative immunoblots. Data are presented as means ± SEM. Statistical significance was determined by unpaired Student's t-test (*, p<0.05; **, p<0.01 for effect of genotype; ^#^, p<0.05; ^##^, p<0.01 for effect of feeding status).
